# Actionable genomic landscape of biliary tract cancer in the Indian population

**DOI:** 10.1093/oncolo/oyaf430

**Published:** 2026-01-10

**Authors:** Sewanti Limaye, Aditya Shreenivas, Darshana Patil, Soumil Vyas, Irene A George, Janani Sambath, Shambhavi Singh, Chetan Madre, Anjali Parab, Pritam Kataria, Darshit Shah, Niyati Shah, Shaheenah Dawood, Nitesh Rohatgi, Ruturaj Deshpande, Aakriti Datta, Humaid Al Shamsi, Andrew Gaya, Ashok Kumar Vaid, Shriniwas Kulkarni, Senthil Rajappa, Damian Rieke, Prashant Kumar, Rajan Datar, Milind Javle

**Affiliations:** Department of Medical and Precision Oncology, Sir H N Reliance Foundation Hospital and Research Centre, Mumbai 422010, India; Department of Medical Oncology and Therapeutics Research, City of Hope Comprehensive Cancer Centre, Duarte, CA 422010, United States; Datar Cancer Genetics, Nashik, Maharashtra 422010, India; Department of Surgical Oncology, Sir H N Reliance Foundation Hospital and Research Centre, Mumbai 51122, India; Manipal Academy of Higher Education (MAHE), Manipal, Karnataka 576104, India; Institute of Bioinformatics, International Technology Park, Bangalore, Karnataka 560066, India; Manipal Academy of Higher Education (MAHE), Manipal, Karnataka 576104, India; Institute of Bioinformatics, International Technology Park, Bangalore, Karnataka 560066, India; Department of Medical and Precision Oncology, Sir H N Reliance Foundation Hospital and Research Centre, Mumbai 422010, India; Department of Medical and Precision Oncology, Sir H N Reliance Foundation Hospital and Research Centre, Mumbai 422010, India; Department of Medical and Precision Oncology, Sir H N Reliance Foundation Hospital and Research Centre, Mumbai 422010, India; Department of Medical and Precision Oncology, Sir H N Reliance Foundation Hospital and Research Centre, Mumbai 422010, India; Department of Medical and Precision Oncology, Sir H N Reliance Foundation Hospital and Research Centre, Mumbai 422010, India; Department of Medical and Precision Oncology, Sir H N Reliance Foundation Hospital and Research Centre, Mumbai 422010, India; Mediclinic Parkview Hospital, Dubai 51122, United Arab Emirates (UAE); Department of Medical Oncology, Fortis Memorial Research Institute, Gurugram 122002, India; Department of Medical and Precision Oncology, Sir H N Reliance Foundation Hospital and Research Centre, Mumbai 422010, India; Datar Cancer Genetics, Nashik, Maharashtra 422010, India; Founder and Director of the Medical Oncology Service at Burjeel Medical City, 9054, United Arab Emirates; Guy’s and St Thomas’ NHS Foundation Trust, University of London, Great Maze Pond SE1 9RT, London; Department of Medical Oncology and Hematology Medanta the Medicity, Gurugram 122001, India; Department of Medical Oncology Sahyadri Super Speciality Hospital, Pune 411004, India; Basavatarakam Indo-American Cancer Hospital and Research Institute, Banjara Hills, Hyderabad 13353, India; Department of Hematology, Oncology and Cancer Immunology, Campus Benjamin Franklin, Charité-Universitätsmedizin Berlin, Corporate Member of Freie Universität Berlin and Humboldt-Universität zu Berlin, Berlin 13353, Germany; Datar Cancer Genetics, Nashik, Maharashtra 422010, India; Koita Centre for Digital Health—Indian Institute of Technology Bombay, Mumbai 400076, India; Datar Cancer Genetics, Nashik, Maharashtra 422010, India; Department of Gastrointestinal Medical Oncology, Division of Cancer Medicine, MD Anderson Centre in Houston, TX 77030, United States

**Keywords:** gall bladder, cholangiocarcinoma, biliary tract cancer, genomic profiling, Indian population

## Abstract

**Background:**

Biliary tract cancers (BTCs), including gallbladder cancer (GBC) and cholangiocarcinoma (CCA), are rare but aggressive malignancies with distinct molecular landscapes and poor prognoses. Genomic profiling has revealed significant molecular alterations, but the genomic landscape of BTC in the Indian population remains underexplored. This study aims to comprehensively characterize the mutation landscape of BTC in the Indian population.

**Methods:**

A total of 154 BTC cases, including 69 CCA and 85 GBC, were retrospectively analyzed using data collected from various targeted sequencing panels. Somatic mutations, copy number variations (CNVs), and gene fusions in key oncogenic and tumor suppressor genes were identified from these panel reports. Downstream analyses were performed to derive key biological insights, including pathway enrichment and mutual exclusivity and co-occurrence analyses of genomic alterations.

**Results:**

*TP53* was the most frequently mutated gene (53%), followed by *KRAS* (18%), *ARID1A* (9%), *IDH1* (7%), and *PIK3CA* (7%). Recurrent amplifications were observed in *MYC* (12%) and *ERBB2* (9%). Pathway enrichment analysis revealed significant dysregulation in the PI3K-AKT-mTOR, Notch, and Wnt/β-catenin signaling pathways. Notably, *IDH1* mutations were primarily observed in CCA, while *STK11* mutations were exclusive to GBC, highlighting distinct molecular characteristics between the two subtypes. PD-L1-negative tumors exhibited distinct genomic alterations, notably *SMAD4* mutations, which were associated with reduced PD-L1 expression. This loss of *SMAD4*, involved in TGF-β signaling, could impair immune response regulation and facilitate immune evasion.

**Conclusions:**

This study provides a comprehensive molecular profiling of BTCs in the Indian population, revealing key genomic alterations, subtype-specific differences, and associations with immune features. The findings underscore the importance of molecular profiling in guiding personalized treatment strategies.

Implications for Practice This study represents the first genomic profiling of biliary tract cancers (BTCs) in Indian patients, revealing both common and alterations compared with global study. Key mutations, including TP53, KRAS, ERBB2, and IDH1, as well as gallbladder cancer–specific STK11 mutations. highlight actionable target for precision therapy. Importantly, the association between SMAD4 loss and reduced PD-L1 expression suggests a potential biomarker for predicting immunotherapy response. These findings underscore the need for routine molecular profiling in BTC management to enable targeted precision directed therapies and improve outcomes in this high-incidence population.

## Introduction

Biliary tract cancers (BTCs) are rare malignancies accounting for approximately 1% of all cancers globally.^1^ Despite low incidence, BTCs are associated with high mortality, reflecting the aggressive clinical course of the disease and limited treatment options. BTCs represent a heterogenous group of tumors with subtypes including gallbladder cancer (GBC) and cholangiocarcinoma (CCA) arising from the gallbladder and the biliary tree, respectively.^2^ These subtypes exhibit distinct clinical outcomes, including varying patterns of recurrence and prognosis, which necessitate tailored management strategies.^3^

GBC is one of the most lethal and aggressive malignancies in the biliary system. Globally, GBC accounts for a small fraction of cancer incidence (1.3%) but contributes to 1.7% of cancer-related mortality.^4^ India accounts for approximately 10% of the global GBC burden, with a higher incidence in the northern and eastern regions.^5^ Despite recent advancements, its prognosis remains poor, with a 5-year survival rate of less than 5% in advanced stages.^6^ Similarly, though CCA is a rare malignancy accounting for 3% of gastrointestinal cancers, its aggressive nature contributes to significant cancer-related mortality. The majority of CCA cases are diagnosed at advanced stages, with a five-year survival rate of 7–20%.^7^ Together, the aggressive nature and poor prognosis of both GBC and CCA strongly signifies the need for detailed molecular characterization to identify actionable targets for improved clinical management.

Comprehensive genomic profiling of BTCs has revealed distinct molecular landscapes across its subtypes. Studies utilizing next-generation sequencing (NGS) have identified key driver alterations, including *IDH1*, *BAP1*, *PBRM1*, and *FGFR2* fusions in intrahepatic cholangiocarcinoma; *KRAS*, *CDKN2A*, and *BRCA1* mutations in extrahepatic cholangiocarcinoma; homologous recombination repair deficiency (HRD) genetic mutations and *ERBB2* amplifications in GBC.^8^ Integrated genomic analysis by Pandey et al. explored the mutational landscape of GBC across the Korea, Indian, and Chilean population. This study identified novel significantly altered genes, including *ELF3, EHF, CTNNB1, APC, NSD1, KAT8, STK11, and NFE2L2*, which may play a key role in GBC tumorigenesis.^9^ Additionally, *ERBB2/ERBB3* amplifications and *MET* and *PIK3* alterations have emerged as potential therapeutic targets in GBC, particularly in high-incidence regions like India.^10^ These findings highlight the molecular heterogeneity between BTC subtypes and underscore the importance of genomic profiling in identifying actionable targets, paving the way for precision medicine approaches in BTC management.

Traditionally, systemic treatment for advanced BTC was limited to chemotherapy, offering modest survival benefits. However, recent advancements in immunotherapy and targeted therapies have transformed the treatment landscape for advanced biliary tract cancers, leading to a more personalized approach.^11^ The incorporation of molecular profiling into clinical practice has facilitated the development of precision medicine approaches, allowing for the use of FGFR inhibitors for tumors with *FGFR2* fusions, IDH1 inhibitors for *IDH1*-mutant tumors, and HER2-targeted therapies for *ERBB2*-amplified tumors.^12^ Despite these insights, the genomic landscape of BTC in the Indian population remains underexplored, necessitating comprehensive studies to identify clinically relevant alterations and their impact on prognosis and treatment strategies. The availability of targeted therapies and immunotherapies has increased the demand for comprehensive molecular profiling to guide personalized treatment decisions.

Through comprehensive analysis of 154 BTC cases, we delineate subtype-specific genomic differences, mutation profiling, dysregulated pathways, and PD-l1 expression, collectively highlighting new therapeutic opportunities for improved cancer management.

## Methodology

A total of 154 histologically confirmed BTC cases, comprising 69 CCA and 85 GBC, underwent targeted comprehensive genomic profiling to characterize somatic alterations. The tumor samples were processed, and targeted next-generation sequencing (NGS) was performed using a customized cancer gene panel to detect single nucleotide variants (SNVs), small insertions/deletions (indels), copy number variations (CNVs), and selected gene fusions in clinically relevant oncogenes and tumor suppressor genes. For mutational landscape analysis, the frequency and distribution of somatic mutations were assessed in CCA and GBC separately and combined. Pathway enrichment analysis was conducted using the clusterProfiler R package, with the Hallmark MSigDB database as the reference to identify significantly altered biological pathways (p value < 0.05).

Co-occurrence and mutual exclusivity analyses were performed using the Oncoprint visualization tool in cBioPortal for the study cohort. Gene pairs showing significant mutual exclusivity or co-occurrence were identified, and only those with a q-value < 0.05 (Benjamini–Hochberg–corrected p-values) were selected for further analysis. These identified significant gene pairs were then validated using The Cancer Genome Atlas (TCGA)-BTC dataset. For each pair, survival analysis was performed in the TCGA-BTC cohort with the cBioPortal survival module, comparing cases with co-occurring alterations to those without alterations in both genes.

Additionally, PD-L1 expression status was evaluated in a subset of cases, and genomic alterations were compared between PD-L1-positive and PD-L1-negative tumors to assess potential immune-related biomarkers. PD-L1 expression was assessed by immunohistochemistry (IHC) using either the 22C3 or 28-8 antibody clones, following standardized protocols. Expression was quantified using the Tumor Proportion Score (TPS), and a TPS score of >1% was considered PD-L1 positive, consistent with established clinical cutoffs. Fisher’s exact test was used to compare genomic alteration frequencies between PD-L1-positive and PD-L1-negative groups. The study also examined gender-specific genomic alterations and their distribution across BTC subtypes. To contextualize these findings, the results were compared with publicly available data from the TCGA dataset.

## Results

### Mutation landscape of biliary tract cancer in the Indian population

**Figure 1.**
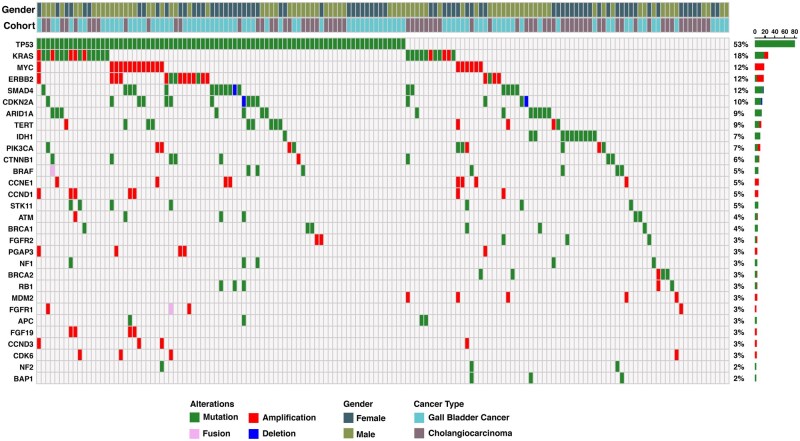
Oncoprint depicting genomic alterations in biliary tract cancer cases. Each column represents an individual tumor sample, while rows correspond to frequently altered genes. The top bar indicates tumor types and Gender, while the side bar denotes mutation frequency across the cohort.

A cohort of 154 BTC cases, comprising 69 CCA and 85 GBC (Table S1), underwent comprehensive genomic profiling to identify somatic mutations, copy number variations (CNVs), and gene fusions. Genomic data were derived from clinical genomic testing conducted using various gene panels. This integrated dataset enabled a comprehensive characterization of the mutational landscape in BTC within the Indian population (Figure 1). Among single nucleotide variants (SNVs), *TP53* (53%) was the most frequently mutated gene, followed by *KRAS* (18%), *ARID1A* (9%), *IDH1* (7%), and *PIK3CA* (7%). *TP53* mutations were prevalent in both GBC and CCA, whereas *IDH1* mutations were primarily observed in CCA. Recurrent *TP53* hotspot mutations, including p. R175H and p. R249S/M, were identified in three cases each. These hotspot mutations occur within the DNA-binding domain, disrupting tumor suppressor function of *TP53* and contributing to oncogenesis.^13^ Copy number analysis revealed recurrent amplifications in *MYC* (12%), *ERBB2* (9%), *CCND1* (5%), and *CCNE1* (5%). Gene fusions were identified in six samples, with *FGFR1* fusions detected in two cases.

**Figure 2.**
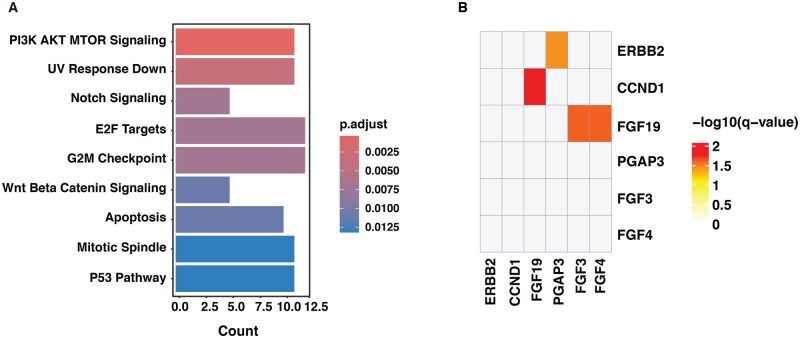
A. Oncogenic pathways enriched in biliary tract cancer. The x-axis denotes the number of altered genes per pathway, while the y-axis lists the significantly enriched pathways. B. Heatmap illustrating co-occurring genomic alterations.

Pathway enrichment analysis of the altered genes revealed significant dysregulation in multiple oncogenic pathways. The most prominently enriched pathways included PI3K-AKT-mTOR signaling, Notch signaling, E2F targets, G2M checkpoint, Wnt/β-catenin signaling, and apoptosis regulation (Figure 2A). The enrichment of these pathways suggests potential molecular targets for therapeutic intervention in BTC. Co-occurring and mutually exclusive analysis revealed significant patterns of amplification co-occurrence. We observed frequent co-occurrence in *ERBB2* and *PGAP3*, *CCND1* and *FGF19, FGF19* and *FGF4*, and *FGF19* and *FGF3* (Figure 2B). To validate these findings, we examined the TCGA BTC dataset, which similarly showed significant co-occurrence of *CCND1* and *FGF19, FGF19* and *FGF4*, and *FGF19* and *FGF3*. Furthermore, survival analysis of TCGA patients with these co-occurring amplifications indicated poorer overall survival compared to patients without any such events (Figure S1—see online supplementary material for a color version of this figure). This suggests that these co-amplifications may have a synergistic role in driving tumor progression in BTC.

### Comparison of genomic alterations between gallbladder cancer and cholangiocarcinoma

**Figure 3.**
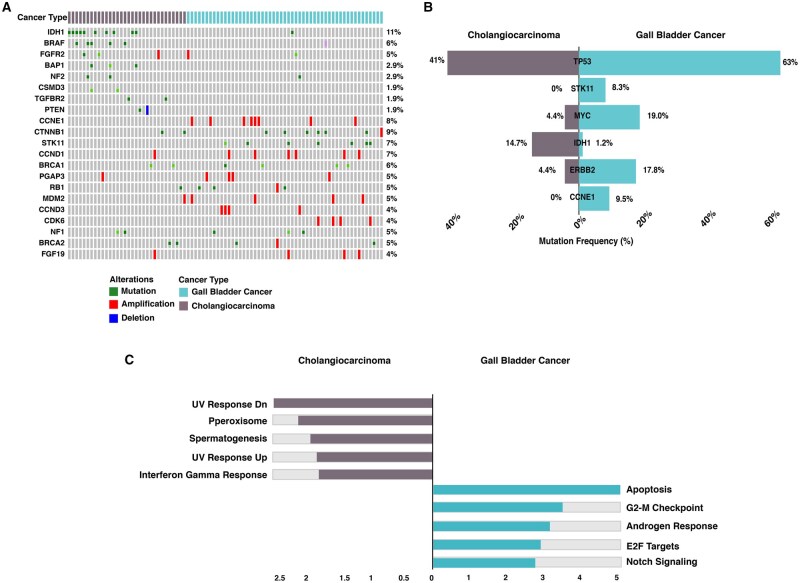
A. Heatmap depicting the top frequently mutated genes in CCA and GBC cohorts. B. Cobar plot depicting genes that are significantly altered between CCA and GBC cohorts. C. Bar graph showing the significantly enriched pathways based on the genes that are uniquely altered in cholangiocarcinoma and gallbladder cancer cohort.

The comparative analysis of genomic alterations between GBC and CCA revealed distinct molecular profiles (Figure 3A and B). *TP53* mutations were the most prevalent across both subtypes, with a higher frequency in GBC, while *IDH1* mutations were predominantly observed in CCA, reinforcing its established role in cholangiocarcinoma tumorigenesis. Additionally, *CCNE1* and *MYC* amplifications were frequently altered in GBC, whereas *STK11* mutations were uniquely identified in GBC. These findings highlight distinct molecular mechanisms driving tumorigenesis in BTC subtypes. Understanding these differences is critical for tailored therapeutic approaches in BTC. Pathway enrichment analysis based on frequently mutated genes in GBC and CCA revealed enrichment of distinct signaling pathways in each subtype. In CCA, UV response dysregulation was enriched, reflecting its association with DNA repair defects, apoptosis resistance, and genomic instability. Additionally, the peroxisome pathway was enriched, indicating its role in cellular metabolism and oxidative stress regulation. In contrast, GBC exhibited enrichment in cell cycle regulation, apoptosis, and Notch signaling, highlighting key mechanisms contributing to tumor progression in this subtype (Figure 3C).

Further analysis of gender-specific genomic alterations in BTC revealed distinct mutational patterns in CCA and GBC. In CCA, IDH1 mutations were more frequent in females (8/29) than males (2/40), and BRAF mutations occurred in 5 of 29 female cases compared to 1 of 40 male cases. In GBC, *RB1* mutations were detected exclusively in females (4/46), whereas *CDK6* alterations were found only in males (4/39), indicating possible differences in cell cycle dysregulation between genders. Comparison with the TCGA BTC dataset did not reveal similar gender-specific trends, suggesting that these observations may be specific to the Indian cohort. The differences in genomic alterations between males and females highlight the need for further investigation into potential gender-based tumorigenic mechanisms and therapeutic implications (Figure S2—see online supplementary material for a color version of this figure).

### PD-L1 status in biliary tract cancer

PD-L1 expression was evaluated in 63 BTC cases, revealing PD-L1 positivity in 14 cases (19.1%) and PD-L1 negativity in 49 cases (80.8%). Among the PD-L1 positive cases, 7 were from CCA and 7 were from GBC. In the PD-L1-negative group, 29 cases were CCA, and 20 cases were GBC. Further analysis of PD-L1-negative tumors identified key genomic alterations associated with this subgroup.

Frequency of *SMAD4* mutations was found higher in PD-L1-negative cases (11/49; 22.4%) compared to PD-L1-positive cases (2/14; 14.3%). Although the difference was not statistically significant (Fisher’s exact test, *P* = .71), recurrent *SMAD4* alteration in the PD-L1-negative subtype may reflect defects in TGF-β signaling, a pathway known to influence immune regulation and tumor progression. Similarly, *ARID1A* mutations (14.3%) and *MYC* amplifications (10.2%) occurred only in PD-L1-negative tumors (*P* = .33 and *P* = .58), suggesting a pathway dysregulation contributing to immune evasion.

## Discussion

Biliary tract cancers are a heterogenous group of aggressive tumors with a poor prognosis and a 5-year survival of 2% in metastatic settings.^2^ The incidence of the cancer is increasing, and the majority of the tumors are diagnosed at advanced stages of the disease.^14^ Molecular profiling of BTCs has emerged as a critical tool for identifying actionable alterations, subclassifying tumors, and enabling personalized therapeutic strategies. However, the lack of large-scale genomic data from the Indian cohort limits the translation of global findings to this high-incidence population. Here, we present data from a clinically annotated cohort of 154 BTC analyzed using a comprehensive molecular profiling panel, comprising 69 CCA and 85 GBC.

The targeted sequencing analysis identified *TP53* as the most frequent alteration in our cohort, followed by *KRAS, ERBB2, MYC*, and *CDKN2A*. Real-world data from 454 BTC samples in the Tempus database showed similar frequent alterations, including *TP53* (42.5%), *CDKN2A* (23.4%), *ARID1A* (19.6%), *BAP1* (15.5%), and *KRAS* (15%).^15^ Compared to the Tempus database, our data revealed a similar pattern of frequent alterations, with some overlapping key mutations and a few distinct variations. Comparative analyses across different populations reveal both overlapping and region-specific mutation profiles. In the Chinese population, the five most frequently mutated genes were *TP53, KRAS, ARID1A, PBRM1*, and *SMAD4*, whereas in the Western population, *IDH1, ARID1A*, *BAP1, TP53*, and *KRAS* were frequently mutated.^16^ Moreover, *IDH1* and *FGFR2* alterations were more frequent in intrahepatic cholangiocarcinoma (iCCA) samples from Western populations, whereas *KRAS*, *SMAD4*, and *ERBB2* mutations were prevalent in Eastern populations.^17^

Several of these recurrent genetic alterations, most notably TP53, KRAS, and ERBB2, have demonstrated significant associations with disease progression and clinical outcomes in BTCs*. TP53,* the recurrently mutated gene in our cohort, is a well-characterized tumor suppressor and is frequently altered across multiple cancer types. BTC patients with *TP53* mutations are reported to have shorter progression-free survival (PFS).^18^  *KRAS* variants are found to be 20% to 30% of BTCs and are associated with aggressive phenotypes and shorter survival.^19–21^ Similarly, *ERBB2* amplifications in BTCs have been associated with shorter PFS and overall survival (OS) in the absence of ERBB2-targeted therapy.^22^ These findings underscore the importance of molecular profiling in identifying high-risk patients and guiding therapeutic interventions tailored to the underlying genetic alterations.

The mutual exclusivity and co-occurrence analysis revealed co-occurrence of gene pairs such as *CCND1-FGF19, FGF19-FGF3, FGF19-FGF4*, and *ERBB2-PGAP3*. Notably, *CCND1, FGF3, FGF4*, and *FGF19* are part of an amplicon located on chromosome 11q13, and these co-occurrences have been previously reported.^23–25^ Furthermore, in lung cancer, the co-amplification of *CCND1* along with *FGF19* promotes cell proliferation, highlighting its critical role in oncogenic signaling and therapeutic resistance.^23^ In addition, co-amplification and co-expression of *PGAP3* with *ERBB2* have been associated with advanced disease features and poor survival in gastric cancer, highlighting its prognostic significance.^26^ The co-occurrence of these genes and their involvement in oncogenic signaling suggest a possible role in conferring an aggressive phenotype in BTCs.

Subtype-based analysis of genomic alterations between CCA and GBC revealed *STK11* and *CCNE1* to be uniquely altered in GBC, highlighting potential cancer type-specific genomic features. *STK11* alterations have been reported in GBC and are associated with reduced OS, PFS, and resistance to immunotherapy.^27–29^ In contrast, *IDH1* mutations were more commonly observed in CCA than in GBC in our cohort and are implicated in promoting cell proliferation, migration, and invasion.^30^ Mutant IDH1 induces epigenetic reprogramming and metabolic alterations that promote oncogenesis in CCA, establishing it as a key driver of tumorigenesis.^31^ The therapeutic relevance of IDH1 alterations has been clinically validated in the ClarIDHy phase III trial. The ClarIDHy trial evaluated the efficacy of the IDH1 inhibitor ivosidenib (AG-120) in unresectable or metastatic CCA patients with *IDH1* mutation.^32^ The study demonstrated a significant improvement in progression-free survival and overall survival in patients treated with ivosidenib.^32^^,^^33^ This pivotal trial established *IDH1* as a clinically actionable target in CCA and exemplifies how molecular characterization can directly inform treatment decisions and guide precision oncology strategies. Similar to *IDH1*, *FGFR2* represents another clinically actionable target in CCA. FGFR inhibitors, such as pemigatinib, are approved for advanced or metastatic CCA with FGFR2 fusions, with several other compounds under investigation.^34^ However, *FGFR2* alterations show notable geographic variation, with higher prevalence in the Western region than in the Eastern population.^17^^,^^35^^,^^36^ In our study as well, we detected a low frequency (4%) of *FGFR2* alterations in CCA. Collectively, our subtype-specific findings underscore the unique genomic landscapes of CCA and GBC, highlighting *STK11* and *IDH1* as critical molecular markers with potential implications for prognosis and targeted therapy in the Indian patients.

Further, the immunogenic nature of BTCs has prompted extensive evaluation of immunotherapy as a potential treatment strategy. Recent advances have demonstrated that combining immunotherapy with standard chemotherapy can improve clinical outcomes. For example, the TOPAZ-1 trial showed a significantly improved overall survival in patients treated with durvalumab plus chemotherapy.^37^ The KEYNOTE-966 trial supports the combination of pembrolizumab with gemcitabine and cisplatin as a first-line treatment for advanced BTC, further establishing immunotherapy as a standard therapeutic approach in this setting.^38^ Collectively, these pivotal studies position immunotherapy as an integral component in managing BTC.

**Figure 4.**
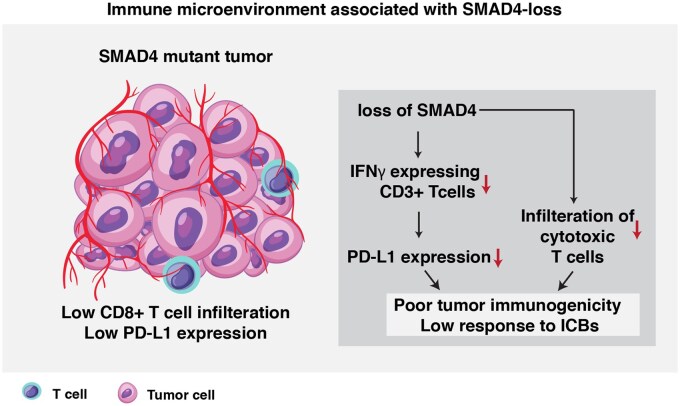
Impact of *SMAD4* loss on the tumor microenvironment and immunotherapy response. *SMAD4* loss in tumors leads to reduced IFNγ-expressing CD3+ T cells, decreased PD-L1 expression, and impaired infiltration of cytotoxic CD8+ T cells into the tumor microenvironment.

Beyond the clinical response, increasing evidence suggests the role of the molecular and genetic landscape in shaping the tumor–immune microenvironment and influencing immunotherapy outcomes. For example, mutations in *IDH1, FGFR2, KRAS, BRAF*, and *HER2* can significantly impact the overall tumor immunogenicity in BTC.^39^ To further understand the relationship between these alterations and immune response, we compared the mutation profiles of PD-L1 positive and PD-L1 negative patients in our cohort, aiming to identify specific mutations associated with immune evasion and potential therapeutic targets for immunotherapy. We observed the higher frequency of *SMAD4* mutation in PD-L1 negative patients. *SMAD4* is a tumor suppressor gene and a key downstream effector of the TGF-β signaling pathway, and its loss has been reported in wide several tumor types.^40^ In pancreatic cancer, *SMAD4* loss is linked to be associated with poor immunogenicity as well as low expression of PD-L1.^40^ The loss of *SMAD4* has been shown to decrease IFNγ-producing T cells, which impacts PD-L1 expression and also leads to decreased infiltration of cytotoxic T cells within the tumor microenvironment.^40^ We observed a similar association between the loss of *SMAD4* and PD-L1-negative patients in our cohort. Figure 4 illustrates a data-driven pathway depicting the low expression of PD-L1 in this subset of patients. However, the underlying mechanism requires further validation through *in vitro* models. These alterations result in poor tumor immunogenicity and diminished responses to immune checkpoint blockade therapies, suggesting *SMAD4* could be a potential predictive biomarker for immunotherapy response.

## Conclusions

Our study provides a comprehensive molecular profiling of BTCs, highlighting critical genomic alterations in Indian patients. The mutational landscape revealed alterations in key genes such as *TP53, KRAS, MYC*, and *ERBB2,* and cancer-specific alterations such as the exclusive presence of *STK11* and *CCNE1* mutations in gallbladder cancer and the higher frequency of *IDH1* mutations in cholangiocarcinoma underscore the need for routine molecular testing to guide targeted therapy. We also observed associations between specific genetic alterations and immune features, such as the loss of *SMAD4*, which correlated with reduced PD-L1 expression, providing insights into potential biomarkers for patient stratification. Further validation on a larger cohort and functional validations will aid to translate these findings in clinics.

## Supplementary Material

oyaf430_Supplementary_Data

## Data Availability

Data will be available on the request from the corresponding author.
