# Supplementary material for: Actionable genomic landscape of biliary tract cancer in the Indian population
Source: Oncologist. 2026 Jan 10;31(3):oyaf430. doi: 10.1093/oncolo/oyaf430 (PMC12923113; doi:10.1093/oncolo/oyaf430)
Supplement: oyaf430_Supplementary_Data [file oyaf430_supplementary_data.pdf]

Comprehensive Genomic Profiling of Biliary Tract Cancer in Indian Population

Supplementary Table

Category	GBC	CCA
**Gender**		
Male	39	40
Female	46	29
**Age (years)**		
>=50	67	53
<50	18	16
**PD-L1 Status**		
PD-L1 Positive	7	7
TPS 1	2	4
1< TPS <10	4	3
TPS > 10	1	0
PD-L1 Negative	20	29
**CCA Subtype**		
Intrahepatic (iCCA)	-	30
Extrahepatic (eCCA)	-	4
Unknown	-	35

Supplementary Table 1: The demographic and molecular characteristics of the patients. GBC – Gallbladder Cancer; CCA – Cholangiocarcinoma;

Supplementary Figures

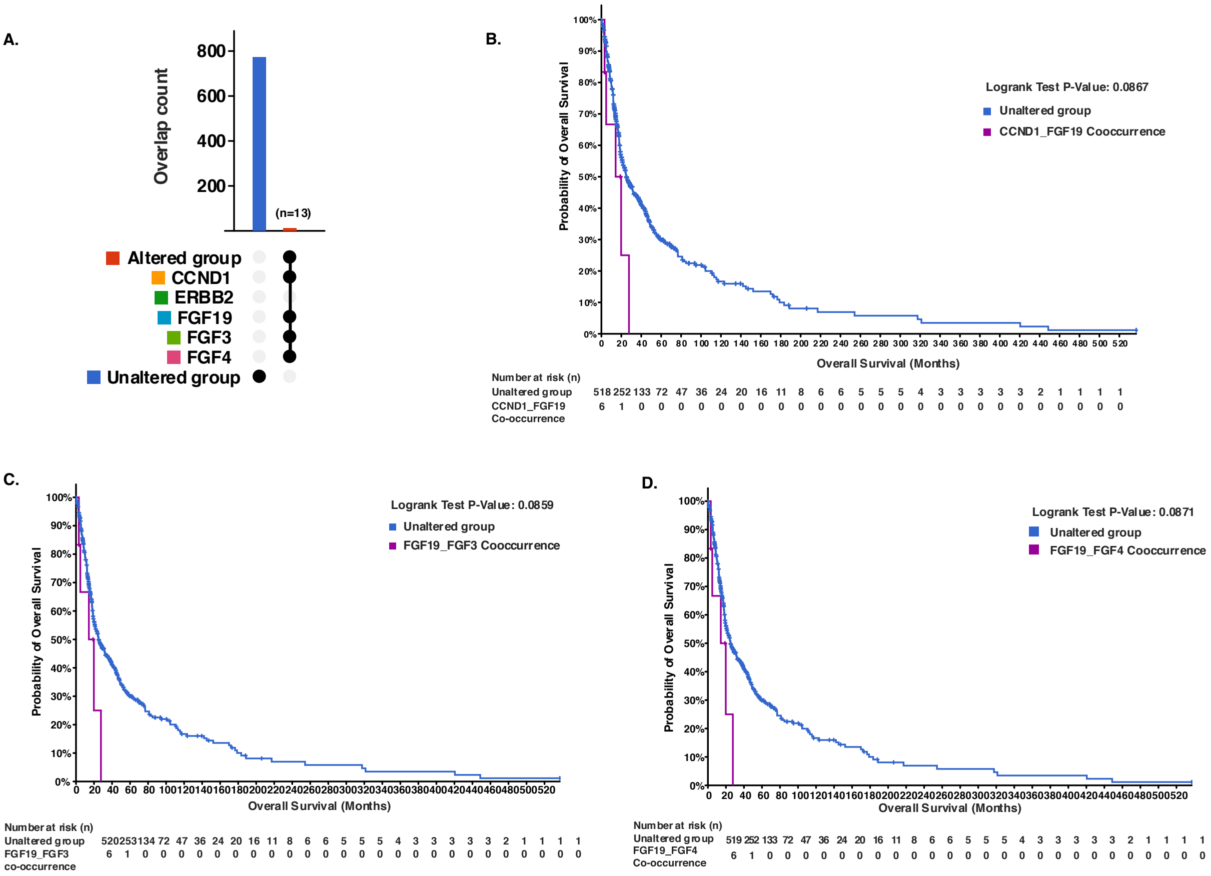

Supplementary Figure 1: (A) Overlap analysis of selected gene amplifications showing the number of patients harboring each co-occurring event. (B-D) Kaplan-Meier survival curves comparing overall survival between patients with the co-occurrence of (B) *CCND1_FGF19*, (C) *FGF19_FGF3*, and (D) *FGF19_FGF4* and the unaltered group.

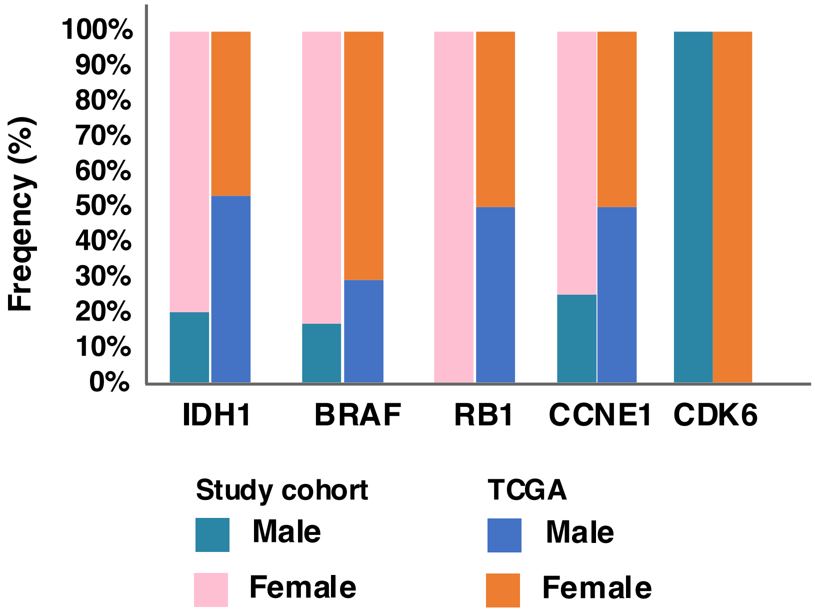

Supplementary Figure 2: A stacked bar plot depicting the mutation frequency of selected genes in male and female cohorts from the study dataset (left bars for each gene) and the TCGA dataset (right bars for each gene).
